# Evaluating virtual role play based learning to improve the confidence and competence of Junior Doctors undertaking on call shifts in inpatient Psychiatry

**DOI:** 10.1192/bjo.2021.391

**Published:** 2021-06-18

**Authors:** Katherine Gardner

**Affiliations:** Surrey and Borders Partnership NHS Foundation Trust

## Abstract

**Aims:**

To enable junior doctors to practice their clinical skills in managing psychiatric emergencies via virtual role plays, and to gain confidence and competence in their skills in acute psychiatry. Lecture based learning about psychiatric emergencies is a part of the induction programme for all junior doctors starting their placements however practical learning and practice of skills in this area is not. The COVID-19 pandemic has further exacerbated this issue by providing an additional challenge to the delivery of face to face teaching for junior doctors both in clinical and educational settings.

**Method:**

The author offered a virtual role play based teaching session to two cohorts of Junior Doctors (GP trainees and foundation trainees) who were starting their psychiatric hospital placements at Surrey and Borders Partnership. The virtual sessions were conducted over Microsoft teams. This session had been run once before as face to face teaching (F2F) in January 2019 (N = 9) prior to the COVID-19 pandemic. Data from this session were compared to data obtained from the virtual sessions in November 2020 and January 2021 (N = 16).

Pre and post study questionnaires were administered via Microsoft Forms. Each session lasted 1 hour and consisted of 3 different role play scenarios based around acute psychiatric emergencies. One junior doctor volunteer acted as the ‘patient’ in each scenario and another volunteer as the ‘doctor’. The other participants all acted as observers. Each scenario lasted 10 minutes with ten minutes for feedback from the researcher afterwards using the ALOBA framework.

Categorical, ordinal data were collected using a Likert scale and general qualitative feedback was also gathered.

**Result:**

The questionnaire return rate was 100% for F2F teaching and 57% for virtual teaching. 100% of participants felt that F2F role play was an acceptable way to practice skills in acute psychiatry vs 75% of participants who felt this about virtual role play. 100% of participants found that F2F role play was ‘quite’ or ‘very’ effective in improving their confidence and perceived competence in acute psychiatry vs 88% of participants who felt this about virtual role play.

**Conclusion:**

Virtual role play based learning is an acceptable and effective method in improving the confidence and perceived competence of junior doctors undertaking on call shifts in inpatient psychiatry but it appears to be less effective than face to face role play based learning. The researcher will act upon the qualitative feedback obtained which suggested ways in which the virtual session could be improved.

